# SMART: A Swing-Assisted Multiplexed Analyzer for Point-of-Care Respiratory Tract Infection Testing

**DOI:** 10.3390/bios13020228

**Published:** 2023-02-04

**Authors:** Li Zhang, Xu Wang, Dongchen Liu, Yu Wu, Li Feng, Chunyan Han, Jiajia Liu, Ying Lu, Dmitriy V. Sotnikov, Youchun Xu, Jing Cheng

**Affiliations:** 1 Department of Biomedical Engineering, School of Medicine, Tsinghua University, Beijing 100084, China; 2 CapitalBiotech Technology, Beijing 101111, China; 3 National Engineering Research Center for Beijing Biochip Technology, Beijing 102200, China; 4 A.N. Bach Institute of Biochemistry, Research Center of Biotechnology of the Russian Academy of Sciences, Leninsky Prospect 33, 119071 Moscow, Russia

**Keywords:** LAMP, point-of-care, SARS-CoV-2, microfluidics, multiplexed detection

## Abstract

Respiratory tract infections such as the ongoing coronavirus disease 2019 (COVID-19) has seriously threatened public health in the last decades. The experience of fighting against the epidemic highlights the importance of user-friendly and accessible point-of-care systems for nucleic acid (NA) detection. To realize low-cost and multiplexed point-of-care NA detection, a swing-assisted multiplexed analyzer for point-of-care respiratory tract infection testing (SMART) was proposed to detect multiple respiratory tract pathogens using visible loop-mediated isothermal amplification. By performing hand-swing movements to generate acceleration force to distribute samples into reaction chambers, the design of the SMART system was greatly simplified. By using different format of chips and integrating into a suitcase, this system can be applied to on-site multitarget and multi-sample testing. Three targets including the N and Orf genes of SARS-CoV-2 and the internal control were simultaneously analyzed (limit of detection: 2000 copies/mL for raw sample; 200 copies/mL for extracted sample). Twenty-three clinical samples with eight types of respiratory bacteria and twelve COVID-19 clinical samples were successfully detected. These results indicate that the SMART system has the potential to be further developed as a versatile tool in the diagnosis of respiratory tract infection.

## 1. Introduction

Respiratory tract infections pose a major global health challenge to the public worldwide, and are associated with high morbidity and mortality [1]. The annual healthcare burden caused by respiratory pathogen infections is enormous globally [2,3]. Accurate and rapid molecular diagnosis of pathogens is of great importance for the subsequent rational treatment, while the point-of-care (POC) system for nucleic acid (NA) detection is urgently needed to rapidly diagnose acute respiratory diseases [4,5]. As a typical acute respiratory disease outbreak in late 2019, coronavirus disease 2019 (COVID-19) has rapidly swept the globe and profoundly changed the world’s medical landscape. To date, the most effective strategy to fight against the COVID-19 pandemic is NA testing and social control to break the chains of transmission. Compared to the standard NA testing technologies, POC systems eliminate the needs of sample transportation, trained operators, and separated clean rooms, and thus greatly accelerate the efficiency of on-site detection of severe acute respiratory syndrome-coronavirus 2 (SARS-CoV-2), as well as other pathogens.

Many well-established commercial POC products have been further developed to detect SARS-CoV-2 and have obtained emergency use authorizations (EUAs) by the Food and Drug Administration (FDA) in the USA [6]. Among these products, the GeneXpert system from cepheid and the FilmArray system from BioFire are the most commonly used POC systems, and can detect the currently prevalent SARS-CoV-2 within 30–60 min in a sensitive and automated manner using reverse transcription-polymerase chain reaction (RT–PCR). However, the cost of these POC products is relatively high and thus hinders their wide application in clinic. An alternative that reduces the cost of the supporting analyzer involves the replacement of PCR with isothermal amplification (IA). Recently, many IA technologies, such as recombinase polymerase amplification (RPA), loop-mediated isothermal amplification (LAMP), and nuclear acid sequence-based amplification (NASBA), have been utilized in the POC systems to facilitate the rapid and low-cost detection of pathogens. The outbreak of COVID-19 also greatly promoted the development of IA-based POC systems for SARS-CoV-2 detection. For example, a MI-IF-RPA assay [7] and a similar centrifugal microfluidic chip [8] were proposed to realize RPA-based NA amplification and lateral flow strip-based result readout. However, the RPA reagent should be prepared off-chip, and only one target can be detected with a strip on these chips, which hinders their practicability. The choice of lyophilized reaction reagent and multiple reaction units is a good solution to overcome these limitations [9], but it is difficult to sensitively detect more targets owing to the limitation of multiplexed RPA [10]. More researchers have attempted to fabricate LAMP-based POC systems, for the low-cost detection of SARS-CoV-2 [11,12], but most of these systems are based on in-tube RT-LAMP or in-tube RT-LAMP followed by CRISPR/Cas reactions [13,14,15,16,17,18,19]. Therefore, the abovementioned shortages regarding reagent preparation or multiplexity are not fundamentally changed. The utilization of microfluidic chips is an effective solution to realize multiplexed and highly automated detection of multiple pathogens or multiple fragments of SARS-CoV-2 [20,21,22,23,24,25,26,27]. In these microfluidic systems, the distribution of samples into separated reaction chambers pre-spotted with primers is an essential prerequisite. A servo or motor is needed for fluid control, and thus, the structure of the supporting analyzer is relatively sophisticated. Therefore, constructing a low-cost microfluidic system with compact size and powerful capability for multiplexed detection is still an unaddressed issue for the POC diagnosis of infectious diseases.

Inspired by our previous studies [28,29] that used a “sine”-shaped channel to realize centrifugation-assisted sample distribution, we proposed a hand-swing microfluidic chip that can realize sample distribution by a hand-swing motion in this study. Since no motor is used, the supporting analyzer is quite simple in structure, and thus, its cost and size are significantly reduced compared to those of the existing POC analyzers. More importantly, this microfluidic system can be flexibly adjusted to satisfy different applications for LAMP-based multitarget or multi-sample detection. Two types of applications were chosen to validate this system. For SARS-CoV-2 detection, a microfluidic chip was designed to detect three targets, including the N and O genes of SARS-CoV-2 and the internal control (IC), and 12 COVID-19 clinical samples could be successfully detected on our SMART system. For multiple bacterial detection, another microfluidic chip was designed to detect eight respiratory bacteria including *Staphylococcus aureus*, *Streptococcus pneumoniae*, *Klebsiella pneumoniae*, *Haemophilus influenzae*, *Acinetobacter baumannii*, *Pseudomonas aeruginosa*, *Stenotrophomonas maltophilia* and *Legionella pneumophila*, and consistent results were obtained for 23 clinical samples compared to the results detected by a commercial LAMP-based bacterial detection kit. To further facilitate field-deployable detection of pathogens, a suitcase with six portable analyzers installed inside was constructed. This suitcase can analyze 36 SARS-CoV-2 samples in parallel in a convenient and flexible manner, and thus possesses potential as a tool to fight against pandemics.

## 2. Materials and Methods

### 2.1. Reagents and Devices

SARS-CoV-2 pseudovirus was commercially purchased from Fubio Biological Technology Corporation (FNV-2019-ncov-abEN, Suzhou, China). The pseudovirus was quantified using digital droplet PCR (ddPCR, TargetingOne, Beijing, China). The sample releasing reagent was obtained from Sansure Biotech (S1014, Changsha, China). A total of 20 ng human genomic DNA (CW0565, Cwbio Biotech, Jiangsu, China) was added as a standard IC of each LAMP reaction. Disposable swabs were purchased from MaiRuiKeLin Technology (93050D, Shenzhen, China). For SARS-CoV-2 RNA extraction, a self-developed lysis-binding buffer from our group [10] was used to purify the viral NAs. For NA extraction of respiratory bacteria, a commercial kit (360090, CapitalBiotech, Beijing, China) was used. Elution buffer was ordered from Qiagen (19086, Beijing, China). Pressure-sensitive adhesive tape for chip encapsulation and inlet/out sealing was provided by Adhesive Research (ARcare^®^7759, Beijing, China).

RT-LAMP was performed in a 20 μL reaction mixture containing 1.57 μL Isothermo buffer (Mg^2+^ free) (A3805B, HaiGene Biotech, Harbin, China), 6.25 mM MgSO_4_ (M3409, Sigma Aldrich, Shanghai, China), 1.175 mM dNTP mixture (B500055-0005, Sangon, Beijing, China), 0.8 μL Bst Enzyme Mix (UDG plus) (13762ES80, Yeasen Biotech, Shanghai, China), 0.12 mM EBT (858390, Sigma Aldrich, Shanghai, China), and LAMP primer mixture (0.2 μM for F3 and B3, 1.6 μM for FIP and BIP, 0.8 μM for LF and LB). The primers were synthesized by Sangon (Beijing, China), and their sequences are listed in Table S1. The EvaGreen^®^Dye was ordered from Biotium (31000, Beijing, China). In-tube RT-LAMP was performed on a 7500 Fast Real-Time PCR System (Thermo-Fisher, Shanghai, China). For on-chip LAMP, the RT-LAMP buffer was lyophilized by SP Scientific Lyophilizer (VisTis Wizard 2.0, Shanghai, China). The RT-LAMP reaction for respiratory bacteria detection with a commercial kit was performed on an RTisochip^TM^—A Analyzer (CapitalBiotech, Beijing, China). The DL 2000 DNA marker and GeneGreen dye were purchased from Tiangen Biotech (Beijing, China). Agarose gel images were obtained and processed using a gel imager (C150, Azure Biosystems, Dublin, CA, USA).

### 2.2. Microfluidic Chip Design and Fabrication

The structure of the chip was designed by following a similar principle as that in our previous studies [28,29]. There are several units for multi-sample detection, in which the number of reaction chambers is adjustable. For each unit, a “sine”-shaped sample-infusing channel is located upward with inlet and outlet at its two ends. The reaction chambers are located downward and are connected with the sample-infusing channel through the connecting channels. The chip was machined by polymethylmethacrylate (PMMA) with a thickness of 3 mm (Figure S1a). Each reaction chamber was 2 mm in depth with a final volume of 20 μL to mimic a standard LAMP reaction in an EP tube, and each “valley” of the sample-infusing channel was designed with a volume larger than 20 μL to ensure that each reaction chamber could be fully filled after sample distribution (Figure S1b). In addition, a handheld area with several grooves was set on the top of the chip to ensure steadiness in the hand-swing process for chip use. In addition, grooves were made on the handheld area of the chip to increase the friction during the hand-swing process.

Prior to use, the chips were ultrasonically cleaned with ethanol and ultrapure water for 30 min sequentially. Then, high-pressure nitrogen was used to blow off the moisture in the chip chamber. The primer pairs and EBT liquid required for the corresponding RT-LAMP reaction target were pre-filled into the reaction chamber, and dried at room temperature for 1 h. Afterward, the lyophilized reagent beads for RT-LAMP (an enzyme bead and a buffer bead for one reaction chamber) were carefully placed into the reaction chambers under low humidity and electrostatic elimination conditions (Figure S1c). Finally, the engraved side of the chip was sealed by pressure-sensitive adhesive (PSA) manually. The inlets/outlets were the also blocked by PSA to obtain a fully sealed chip and prevent lyophilized beads from getting wet and stored at 4 °C before use.

### 2.3. Analyzer Construction

The structure of the supporting analyzer is shown in Figure S2. The shell of the analyzer with a movable cover was 3D printed by black resin. A copper plate (60 × 40 mm^2^) was placed behind the chip for contact heating. A polyimide-heating membrane (12 V, 5 W, Hongxin, Shenzhen, China) was adhered to the back of the copper plate with an NTC temperature sensor (WZPT-10X, SENXTE, Jiangsu, China), which was inserted to measure the temperature. Two flat springs were fixed on this copper plate to ensure that the contact between the chip and the copper was tight when the chip was inserted into the analyzer (Figure S2b). A camera (WXSJ-H65HD, Weixin, Shenzhen, China) was placed in front of the chip to perform visible detection under the illumination of a white light LED plate (Yingxin, Shenzhen, China). Temperature, image capture, and signal transmission were controlled using a control module (JLC, Shenzhen, China). The control module is designed using a STM32 microcontroller (STM32F103C8T6, STMicroelectronics, Geneva, Switzerland), a driver chip (TA6586, Smart-microelectronic, Wuxi, China), and an analog signal reading circuit (INA333, TI, Dallas, Texas, USA) to achieve temperature control, and using a USB port to achieve image capture and signal transmission. The detected signals were transferred to a laptop by a USB port under the monitoring of a software written in Python.

### 2.4. Sample Preparation

To examine the basic performance of in-tube LAMP for SARS-CoV-2, the quantified SARS-CoV-2 pseudoviruses were first mixed in equal proportions with the sample releasing reagent (0.5×) for 10 min to lyse the viral particles at room temperature. Then, the released viral NAs and human genomic DNA were diluted to the known concentrations and detected with LAMP reagents to determine the limit of detection (LoD) for SARS-CoV-2. After that, throat swabs from healthy donors were dipped in 1 mL of rinsing solution (lysis and binding buffer of the kit) to simulate negative clinical samples, while serial dilutions of the SARS-CoV-2 pseudoviruses were immediately added into the rinsing solutions of the swabs to mimic positive clinical samples. All throat swabs were collected more than 30 min after eating or drinking, and the throats of donors were rinsed with water before scraping.

Nine SARS-CoV-2 positive and three negative clinical samples were provided by the CapitalBio MedLab. All clinical samples were collected by clinical nasopharyngeal swabs and stored and transported in VTM following the standard guidelines of the National Health Commission (NHC) of China. To extract the NAs from SARS-CoV-2 mock and clinical samples, all procedures were performed as described in detail in our previous paper [10]. In brief, magnetic beads were added to the 1 mL mock sample and incubated for 5–10 min in a 1.5 mL tube. Then, a magnetic stand was used to adsorb the magnetic beads for 3–5 min, and the supernatant was carefully discarded when nearly all the beads were adsorbed. Next, 600 μL of washing solution was gently added into the tube along the wall to rinse the extracted magnetic beads. After that, the NA was eluted in 20 μL elution buffer for subsequent analysis. For extraction-free NA release, the SARS-CoV-2 mock sample was mixed with an equal volume of the NA-releasing reagent (0.5×) for 10 min at room temperature, and then the mixed solution was loaded into the chip as the template to dissolve the lyophilized RT-LAMP reagent. In one detection unit of a 3 × 3 (sample number × target number) hand-swing chip, the loading volume was approximately 80 μL.

To detect the respiratory bacteria, 23 sputum samples with respiratory tract infections were collected. It was necessary that the white blood cell count was greater than 25/low power field, the epithelial cells were less than 10/low power field to be qualified sputum samples, and the sample volume was not less than 0.6 mL in the sample collection tube. Clinical samples were collected with the patients’ informed consent in the Beijing Tsinghua Changgung Hospital in 2021. This research was approved by the Institution Review Board of Tsinghua University (Project No. 20170012, Beijing, China). NA extractions from these clinical sputum samples were performed by following the protocol as recommended by the respiratory bacterial NA detection kit. In brief, the viscous sputum sample was first liquefied with an equal volume of 4% NaOH solution, and then centrifuged at 20,000× *g* for 5 min for bacterial collection. The pellet was then resuspended in 1 mL of washing buffer for washing, and after another centrifugation at 20,000× *g* for 5 min, the supernatant was discarded. Then, 200 μL eluent was added to the tube and resuspended. Next, the sample was transferred to a tube with zirconium beads, and the tube was placed in a 95 °C-metal bath for incubation at 100× *g* for 10 min to mechanically lyse the bacteria. Finally, after the impurities were precipitated by instantaneous centrifugation, the supernatant with bacterial NA was taken as the template for testing. For the commercial RTisochip^TM^—A system test, 20 μL of the thawed amplification reagent at room temperature was mixed well with 34.5 μL of extracted NA sample in a 200 μL tube. Next, 50 μL of the mixture was added to the centrifugal microfluidic chip in the kit, and then the centrifugal chip was placed on the RTisochip^TM^—A system for LAMP amplification and result interpretation. For our SMART system test, a ten-target hand-swing chip was fabricated and used. DEPC water was added to the 100 μL purified NA sample to increase the volume to 250 μL, and then the solution was loaded into our hand-swing chip as the template, and also to dissolve the lyophilized RT-LAMP reagents for NA amplification.

## 3. Results and Discussion

### 3.1. Workflow of the SMART System

**Figure 1:**
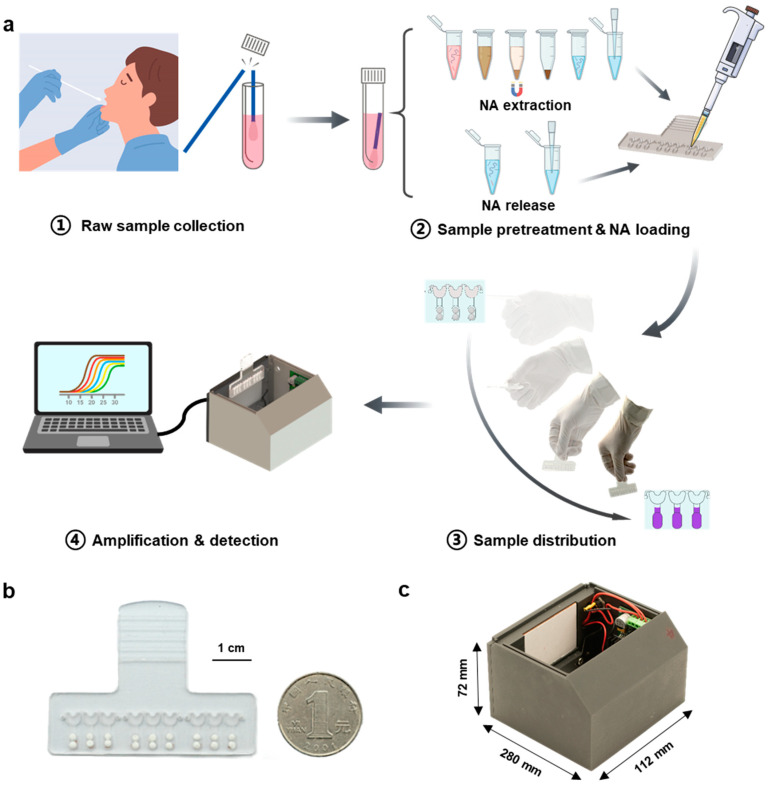
Workflow of the SMART system. (**a**) Four steps for NA testing using the SMART system: ① Nasopharyngeal swab collection, ② NA extraction or release from the swab and NA sample loading into the assembled chip, ③ Quickly swing the chip, and ④ Real-time visual detection of pathogens by inserting the chip into the support analyzer. (**b**) The photograph of an assembled hand-driven chip. (**c**) The photograph of the supporting analyzer.

As shown in Figure 1a, after the raw samples were collected, two strategies were used to treat the samples, including NA extraction and NA release. The former strategy is an essential step in the standard molecular diagnosis, which can be accomplished manually or automatically with the instrument supplied in a clean room, while the latter is simple to execute and is currently widely used in POC systems [26,30,31]. Afterward, a fixed volume of NA sample was loaded into the chip (Figure 1b) to completely fill the sample-infusing channels. The inlet and outlet were then sealed with pressure-sensitive adhesive tape. The operator held the handlebar of the chip and quickly swung 1–3 times. The infused NA sample was evenly distributed into the reaction chambers in the hand-swing process, and the lyophilized LAMP reagents, pre-spotted primers, and EBT indicator were dissolved. The performance of amplification reaction and visual detection on real-time can be accomplished simply by inserting the chip into the supporting analyzer (Figure 1c). The entire system is compact and easy-to-operate, remarkably reducing the reliance on complicated instruments and trained operators.

### 3.2. Chip Structure Optimization

For multitarget detection, the distribution of samples into isolated reaction chambers with respective primers is an important prerequisite. Centrifugation-assisted liquid distribution is a commonly used strategy for multiplexed RPA [32,33], LAMP [22,23] and PCR [28,29]. However, using a centrifuge makes it difficult to further reduce the size and cost of the supporting analyzer. Therefore, construction of a microfluidic analyzer with a compact size and low-cost to facilitate on-site POC applications is a fundamental issue, and substituting the centrifuge with manual operation to generate acceleration for sample distribution is the major innovation in this study.

**Figure 2:**
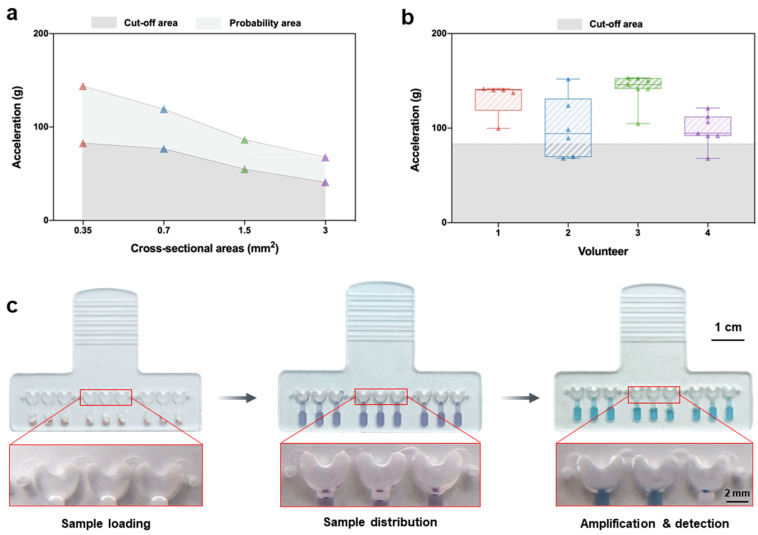
Optimization of the chip structure and characterization of the acceleration for liquid distribution. (**a**) Measurement of acceleration for liquid distribution in connected channels with various cross-sectional areas. The cut-off area (dark gray) indicates that acceleration in this region does not allow the liquid to be completely distributed. The probability area (light gray) indicates that the liquid in this acceleration region has the probability of being distributed. In the acceleration region above these two regions, the liquid could be fully distributed. (**b**) Maximum accelerations generated by four volunteers in several hand-swinging processes. Volunteers 1 and 3 were males, and volunteers 2 and 4 were females. (**c**) Liquid preservation in the chamber of the chip in different states.

To achieve this goal, we fabricated microfluidic chips (Figure 1b and Figure S3) by following the principle for sample distribution in centrifugal microfluidics [28,29]. It was found that the necessary acceleration force for sample distribution is mainly influenced by the size of the connecting channel. Therefore, a series of chips with different sizes of connecting channels were fabricated and tested on a centrifugal platform (Figure S3a) to determine the acceleration needed to achieve uniform liquid distribution. As shown in Figure 2a, the acceleration necessary for fluid distribution was inversely proportional to the cross-sectional area of the connecting channels. Then, the acceleration that could be generated by hand-swinging was measured by four volunteers (two males and two females) with a hand-held acceleration sensor (Figure S4). As shown in Figure 2b, the maximum accelerations generated by four volunteers in several hand-swing processes (Figure S4b and Table S2) were determined. By taking the results shown in Figure 2a,b into consideration, the cross-sectional size of the connecting channel was determined to be 0.5 mm in depth and 1.5 mm in width, to ensure the sample distribution after 1–3 hand-swings (Figure S3b). Although a larger size of this connecting channel made the process of sample distribution easier, it may cause the sample leaking into the reaction chambers in the sample loading stage; thus, a connecting channel with an even larger size was not chosen. After optimizing the chip structure, the chip for visible LAMP was tested with the supporting analyzer. As shown in Figure 2c, after a hand-swing process, the sample in the sample-infusing channel was distributed into the corresponding reaction chambers, and the reaction mixture remained in the reaction chambers after the simulated RT-LAMP process (60 min of incubation at 65 °C). Only a small number of discrete droplets could be observed in the sample-infusing channel due to the evaporation, implying that no liquid connection existed among adjacent reaction chambers. These results demonstrate the feasibility of our hand-swing chip for sample distribution and subsequent LAMP, making the multiplexed detection of various pathogens possible.

### 3.3. Cross-Contamination Evaluation

**Figure 3:**
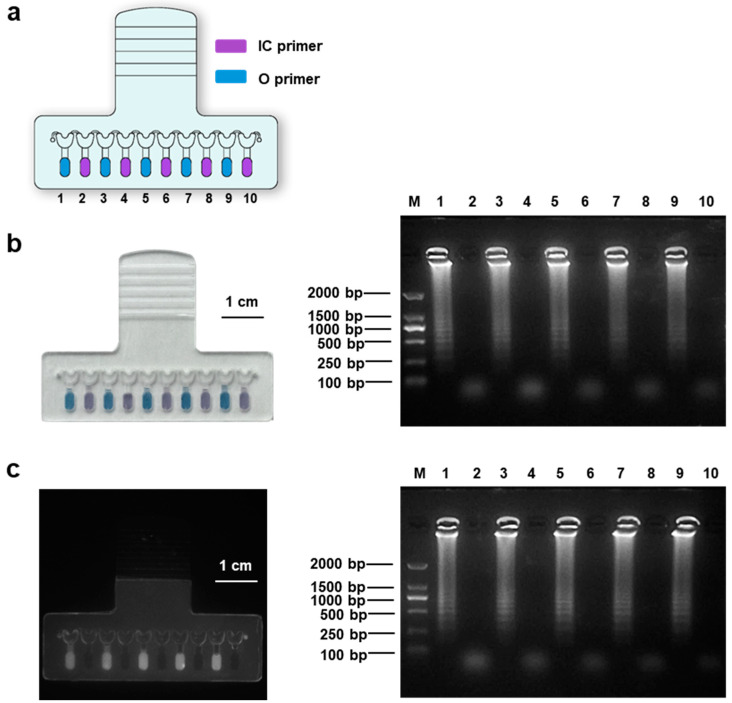
Cross-contamination evaluation based on the EBT-based visible LAMP and EvaGreen-based fluorescent LAMP. (**a**) Schematic diagram of a chip with preloaded primers. The odd-numbered reaction chambers preloaded with primers for the human genome and the even-numbered reaction chambers preloaded with primers for the Orf gene of SARS-CoV-2. (**b**) Photograph of the chip finished with EBT-based visible LAMP amplification (left) and the electrophoretic result of the amplicons (right). The blue color indicates a positive result and the purple color indicates a negative result. (**c**) Photograph of the chip finished with EvaGreen-based fluorescent LAMP amplification (left) under UV and the electrophoretic result of the amplicons (right).

Although there was no observed liquid connection in the sample-infusing channel (Figure 2c), the potential cross-contamination need to be evaluated since the evaporation and condensation of liquid was inevitable during LAMP. Therefore, we preloaded primer pairs for human genomic DNA in odd-numbered reaction chambers and primer pairs for the Orf gene of SARS-CoV-2 in even-numbered reaction chambers (Figure 3a). Then, a 250 μL sample with 100 ng human genomic DNA was infused into the chip for EBT-based visible LAMP. As shown in Figure 3b, only the odd-numbered reaction chambers exhibited a blue color (indicating positive results), while the even-numbered reaction chambers exhibited a purple color (indicating negative results), matching the primer setting (Figure 3a). Furthermore, the amplicons in these reaction chambers were aspirated and assessed by gel electrophoresis; only the amplicons from the odd-numbered reaction chambers showed ladder-like signals, indicating successful DNA amplification, which was not observed for solutions from even-numbered chambers. Similarly, to further demonstrate that no cross-contamination occurred among the adjacent reaction chambers, a similar test was performed by substituting the EBT-based visible LAMP with EvaGreen-based fluorescent LAMP. As shown in Figure 3c, the odd-numbered reaction chambers showed a strong fluorescence signal under UV excitation, while the even-numbered reaction chambers did not, and the gel electrophoresis image showed a similar result as expected. Therefore, no cross-contamination of primers occurred among the adjacent reaction chambers during the LAMP reaction.

### 3.4. Sensitivity for SARS-CoV-2 Detection

**Figure 4:**
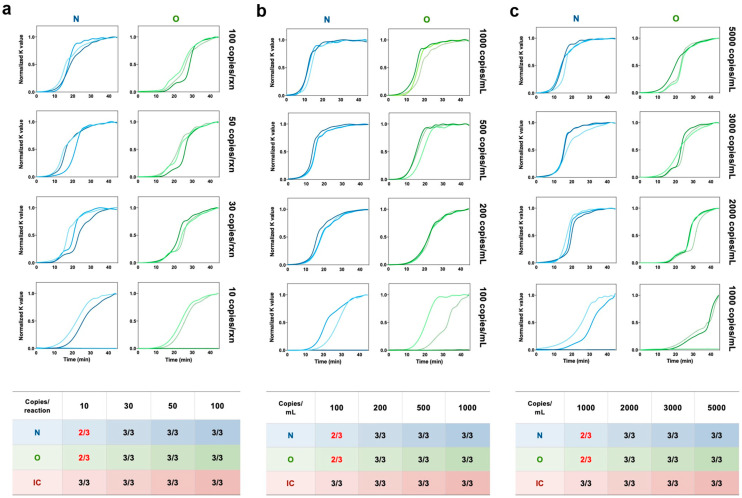
LoD test of our SMART system for SARS-CoV-2 detection. (**a**) Real-time amplification signal of visible RT-LAMP reactions with synthetic SARS-CoV-2 viral RNA at concentrations of 10, 30, 50, and 100 copies/reaction on the chip, respectively. Human genomic DNA (20 ng) was introduced into each reaction chamber. (**b**) Real-time amplification signal of visible RT-LAMP reactions with NA extracted from SARS-CoV-2 mock swab samples with concentrations of 100, 200, 500, and 1000 copies/mL, respectively. (**c**) Real-time amplification signal of visible RT-LAMP reactions with NA directly released from SARS-CoV-2 mock swab samples with concentrations of 1000, 2000, 3000, and 5000 copies/mL, respectively. The table attached summarizes the performance of sensitivity tested with our SMART system. The IC detection results under different concentrations of virus template are listed in Figure S5. Each line in the figure represents an experimental repetition.

Sensitivity is one of the most important indicators for a POC system. Here, we tested the detection limit of our SMART system using serial dilutions of RNA templates of known concentrations released from SARS-CoV-2 pseudoviruses. As shown in Figure 4a and Figure S5, all internal controls of human genomic DNA could be detected well, which means that our visualization reaction system passed the quality inspection. For the SARS-CoV-2 N gene, when the concentration of the virus template was higher than 30 copies/reaction, the reaction could reach the level that the result became visible to the naked eye after 25 min of incubation. Similarly, the SARS-CoV-2 Orf gene could be stably detected after 30 min of incubation at a concentration of 30 copies/reaction.

NA extraction and purification steps are still necessary and effective for robust and sensitive detection. Therefore, the detection limit of the extracted samples from mock swab samples was evaluated using our system. Based on an optimized extraction method proposed in our previous study [10], two targets of SARS-CoV-2 could be stably identified at 200 copies/mL from mock samples with our SMART system (Figure 4b).

Although NA extraction allows sensitive detection, it is time-consuming and sometimes labor-intensive. Direct amplification of samples for POC applications has been frequently reported [26,30,31], so it is therefore necessary to test it on our system. As shown in Figure 4c, our system could achieve extraction-free direct amplification of SARS-CoV-2 at a concentration of 2000 copies/mL. The results were comparable to the results of released NA and extracted NA, and the amplification efficiency was comparable, which demonstrates that our system has excellent compatibility with various types of samples. The above results with three types of NA samples were consistent with the results of the in-tube tests (Figure S6).

### 3.5. Multitarget and Multisample Detection

It is challenging to perform multitarget and multi-sample detections with the current POC systems. For instance, the cartridge from GeneXpert only has one reaction chamber for multiplexed PCR [34], thus the system can only detect several (three to six) targets from one sample simultaneously. An effective strategy to realize multi-sample detection for the GeneXpert system is the combined use of many individually controllable modules [35]; however, this strategy increases the cost of the entire system remarkably. So, it is an optimal choice for our SMART system to further improve its ability for multitarget and multi-sample detection in a low-cost manner.

**Figure 5:**
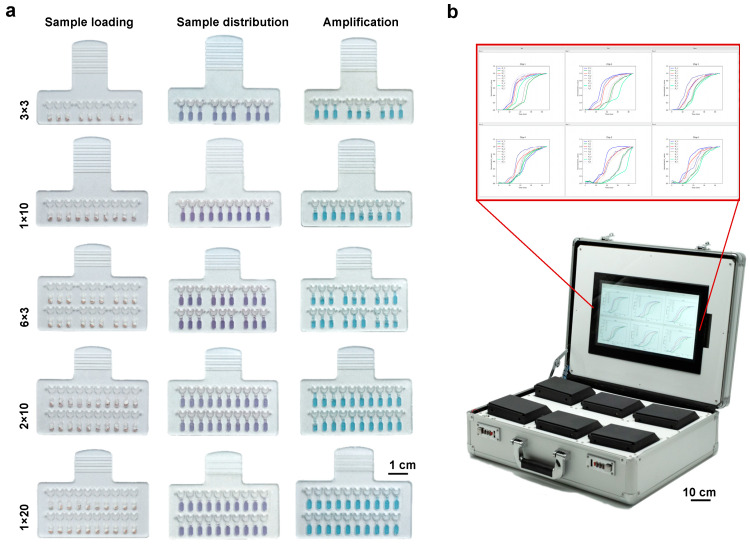
Ability of our SMART system in multitarget and multi-sample detection. (**a**) Illustration of the chips with different designed structures for detecting multiple targets and multiple samples. (**b**) Photograph of the mobile suitcase accommodating six SMART analyzers and the user interface for signal readout.

As shown in Figure 5a, five chips with different patterns have been designed to achieve 3 × 3, 6 × 3, 1 × 10, 2 × 10, and 1 × 20 (sample number × target number) detections to satisfy different requirements. Taking SARS-CoV-2 detection as an example, since only three targets, including the N gene, Orf gene, and IC, need to be tested, up to six samples can be simultaneously analyzed with the 6 × 3 chip. To detect more targets, the 1 × 20 chip allows the simultaneously analysis of 20 targets (including the control). Therefore, our SMART system has much higher flexibility for both multitarget and multi-sample testing.

To further facilitate field-deployable application, a suitcase was constructed to support the work of six SMART analyzers introduced above (Figure 5b and Figure S7). This suitcase contains an industrial computer based on Raspberry Pi to control six analyzers and process the detected signals for read-out (size: 11.2 × 28 × 7.2 cm^3^, weight: 370 g). The systems in the suitcase work with an external power supply or an internal battery, and thus greatly promote the on-site use for fighting epidemics, such as COVID-19.

### 3.6. Detection of Clinical Samples

**Figure 6:**
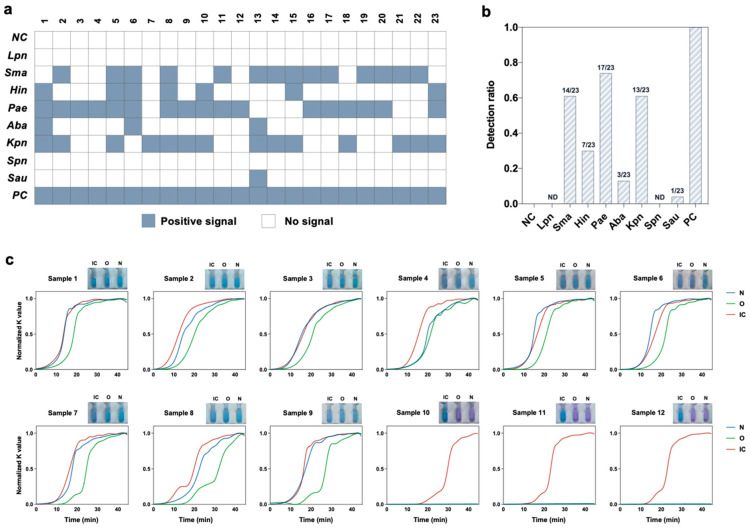
Clinical validation of our SMART system. (**a**) Detection of RNA extracted from human sputum specimens from 23 patients infected with respiratory pathogens. The gray-blue squares represent positive signals. The white squares represent no signal was detected. (**b**) Detection probability statistics of each respiratory pathogen. (**c**) Detection of COVID-19 clinical samples. (Note: ND, not detected; NC, negative control; PC, positive control; IC, internal control. *Sau*, *Staphylococcus aureus*. *Spn*, *Streptococcus pneumoniae*. *Kpn*, *Klebsiella pneumoniae*. *Hin*, *Haemophilus influenzae*. *Aba*, *Acinetobacter baumannii*. *Pae*, *Pseudomonas aeruginosa*. *Sma*, *Stenotrophomonas maltophilia*. *Lpn*, *Legionella pneumophila*).

We collected 23 sputum samples from patients infected with respiratory pathogens, 9 nasopharyngeal swab samples from patients infected with SARS-CoV-2, and 3 negative swab samples to test our SMART system and to demonstrate the ability of our SMART system to analyze real clinical samples (Table S3). The respiratory bacterial results were further confirmed by a commercial kit that was approved by the National Medical Products Administration (NMPA) of China. A consistent result was obtained (Figures S8 and S9), demonstrating the practicality of our SMART system. The detected bacteria for each sample were summarized in Figure 6a. It was found that all 23 samples contained at least two bacteria, indicating that coinfection frequently occurred. Among the eight bacteria detected with our SMART system, *Pseudomonas aeruginosa*, *Stenotrophomonas maltophilia* and *Klebsiella pneumoniae*, which are commonly found pathogens in the sputum of long-stay patients [36], were detected in over 50% of the samples (Figure 6b). In addition, *Streptococcus pneumoniae* and *Legionella pneumophila* were not detected in these samples. Nine COVID-19 positive samples and three negative samples were also successfully detected (Figure 6c). The above results indicate that our system can accurately and simultaneously detect a variety of respiratory pathogens with high fidelity.

## 4. Conclusions

In this study, we proposed an easy-to-use and low-cost SMART system to detect multiple pathogens with EBT-based visible LAMP in a multiplexed manner. An essential step for distributing the sample into reaction chambers was achieved by a hand-swinging process; thus, the structure of the chip and its supporting analyzer is quite simple, making low-cost and flexible detection of multitarget and multiple samples possible (Table S4). This SMART system has been successfully applied for the detection of respiratory pathogens, including SARS-CoV-2. By integrating six analyzers, a compact industrial computer, and rechargeable battery together, a suitcase that can simultaneously detect 36 SARS-CoV-2 samples was constructed, which has the potential to work as a field-deployable laboratory for the on-site diagnosis of respiratory tract infection. Certainly, the SMART system proposed here is suitable to adapt to samples that do not involve complicated pretreatments. For the sample with complex interfering materials, an individual NA-extraction module is preferred to extend the capability of our SMART system. Overall, the SMART system proposed in this study possesses great significance in the low-cost, flexible, and multiplexed detection of pathogens, and could be further developed as a versatile tool for in vitro molecular diagnosis.

## Data Availability

Data are contained within the article. Initial data are available on request from the corresponding author.

## Supplementary Materials

The following supporting information can be downloaded at: https://www.mdpi.com/article/10.3390/bios13020228/s1, Figure S1: Design and assembly of the microfluidic chip; Figure S2: Construction of the analyzer; Figure S3: Optimization of the sizes of the connecting channel on the chip; Figure S4: Statistics and measurement of the acceleration generated by hand-swinging; Figure S5: Real-time visualization of the IC detection results in the on-chip LoD test; Figure S6: In-tube LoD test for SARS-CoV-2 detection; Figure S7: Dimensions and weight of the suitcase equipped with six SMART analyzers; Figure S8: The results of 23 clinical sputum specimens analyzed by the commercial RTisochipTM-A system; Figure S9: The results of 23 clinical sputum specimens analyzed by our SMART system; Table S1: Primer sequence of each target; Table S2: Maximum acceleration (g) per swing in one minute; Table S3: Summary table for the detection of clinical respiratory pathogen samples; Table S4: The cost of the supporting analyzer; Table S5: Comparison of existing molecular diagnostics with SMART system. Supplementary figures and tables associated with this article can be found at the end of article.

## References

[1] WHO The Top 10 Causes of Death. https://www.who.int/news-room/fact-sheets/detail/the-top-10-causes-of-death.

[2] Wilder-Smith A., Osman S. (2020). Public health emergencies of international concern: A historic overview. J. Travel Med..

[3] Wang C., Horby P.W., Hayden F.G., Gao G.F. (2020). A novel coronavirus outbreak of global health concern. Lancet.

[4] Song Q., Sun X., Dai Z., Gao Y., Gong X., Zhou B., Wu J., Wen W. (2021). Point-of-care testing detection methods for COVID-19. Lab Chip.

[5] Valera E., Jankelow A., Lim J., Kindratenko V., Ganguli A., White K., Kumar J., Bashir R. (2021). COVID-19 Point-of-Care Diagnostics: Present and Future. ACS Nano.

[6] FDA Coronavirus Disease 2019 (COVID-19) Emergency Use Authorizations for Medical Devices. https://www.fda.gov/medical-devices/coronavirus-disease-2019-covid-19-emergency-use-authorizations-medical-devices/in-vitro-diagnostics-euas-molecular-diagnostic-tests-sars-cov-2..

[7] Liu D., Shen H., Zhang Y., Shen D., Zhu M., Song Y., Zhu Z., Yang C. (2021). A microfluidic-integrated lateral flow recombinase polymerase amplification (MI-IF-RPA) assay for rapid COVID-19 detection. Lab Chip.

[8] Cao G., Huo D., Chen X., Wang X., Zhou S., Zhao S., Luo X., Hou C. (2022). Automated, portable, and high-throughput fluorescence analyzer (APHF-analyzer) and lateral flow strip based on CRISPR/Cas13a for sensitive and visual detection of SARS-CoV-2. Talanta.

[9] Yin K., Ding X., Li Z., Sfeir M.M., Ballesteros E., Liu C. (2021). Autonomous lab-on-paper for multiplexed, CRISPR-based diagnostics of SARS-CoV-2. Lab Chip.

[10] Liu J., Wang H., Zhang L., Lu Y., Wang X., Shen M., Li N., Feng L., Jing J., Cao B. (2022). Sensitive and Rapid Diagnosis of Respiratory Virus Coinfection Using a Microfluidic Chip-Powered CRISPR/Cas12a System. Small.

[11] Chaouch M. (2021). Loop-mediated isothermal amplification (LAMP): An effective molecular point-of-care technique for the rapid diagnosis of coronavirus SARS-CoV-2. Rev. Med. Virol..

[12] Kang T., Lu J., Yu T., Long Y., Liu G. (2022). Advances in nucleic acid amplification techniques (NAATs): COVID-19 point-of-care diagnostics as an example. Biosens. Bioelectron..

[13] Zhu X., Wang X., Han L., Chen T., Wang L., Li H., Li S., He L., Fu X., Chen S. (2020). Multiplex reverse transcription loop-mediated isothermal amplification combined with nanoparticle-based lateral flow biosensor for the diagnosis of COVID-19. Biosens. Bioelectron..

[14] Xun G., Lane S.T., Petrov V.A., Pepa B.E., Zhao H. (2021). A rapid, accurate, scalable, and portable testing system for COVID-19 diagnosis. Nat. Commun..

[15] Bokelmann L., Nickel O., Maricic T., Paabo S., Meyer M., Borte S., Riesenberg S. (2021). Point-of-care bulk testing for SARS-CoV-2 by combining hybridization capture with improved colorimetric LAMP. Nat. Commun..

[16] Wang R., Qian C., Pang Y., Li M., Yang Y., Ma H., Zhao M., Qian F., Yu H., Liu Z. (2021). opvCRISPR: One-pot visual RT-LAMP-CRISPR platform for SARS-cov-2 detection. Biosens. Bioelectron..

[17] Panpradist N., Kline E.C., Atkinson R.G., Roller M., Wang Q., Hull I.T., Kotnik J.H., Oreskovic A.K., Bennett C., Leon D. (2021). Harmony COVID-19: A ready-to-use kit, low-cost detector, and smartphone app for point-of-care SARS-CoV-2 RNA detection. Sci. Adv..

[18] Ge A., Liu F., Teng X., Cui C., Wu F., Liu W., Liu Y., Chen X., Xu J., Ma B. (2022). A Palm Germ-Radar (PaGeR) for rapid and simple COVID-19 detection by reverse transcription loop-mediated isothermal amplification (RT-LAMP). Biosens Bioelectron.

[19] Ye H., Nowak C., Liu Y., Li Y., Zhang T., Bleris L., Qin Z. (2022). Plasmonic LAMP: Improving the Detection Specificity and Sensitivity for SARS-CoV-2 by Plasmonic Sensing of Isothermally Amplified Nucleic Acids. Small.

[20] Xiong H., Ye X., Li Y., Wang L., Zhang J., Fang X., Kong J. (2020). Rapid Differential Diagnosis of Seven Human Respiratory Coronaviruses Based on Centrifugal Microfluidic Nucleic Acid Assay. Anal. Chem..

[21] Tian F., Liu C., Deng J., Han Z., Zhang L., Chen Q., Sun J. (2020). A fully automated centrifugal microfluidic system for sample-to-answer viral nucleic acid testing. Sci. China Chem..

[22] Xing W., Liu Y., Wang H., Li S., Lin Y., Chen L., Zhao Y., Chao S., Huang X., Ge S. (2020). A High-Throughput, Multi-Index Isothermal Amplification Platform for Rapid Detection of 19 Types of Common Respiratory Viruses Including SARS-CoV-2. Engineering.

[23] Xing W., Wang J., Zhao C., Wang H., Bai L., Pan L., Li H., Wang H., Zhang Z., Lu Y. (2021). A Highly Automated Mobile Laboratory for On-site Molecular Diagnostics in the COVID-19 Pandemic. Clin. Chem..

[24] Li N., Shen M., Liu J., Zhang L., Wang H., Xu Y., Cheng J. (2021). Multiplexed detection of respiratory pathogens with a portable analyzer in a "raw-sample-in and answer-out" manner. Microsyst. Nanoeng..

[25] Soares R.R.G., Akhtar A.S., Pinto I.F., Lapins N., Barrett D., Sandh G., Yin X., Pelechano V., Russom A. (2021). Sample-to-answer COVID-19 nucleic acid testing using a low-cost centrifugal microfluidic platform with bead-based signal enhancement and smartphone read-out. Lab Chip.

[26] Nguyen H.Q., Bui H.K., Phan V.M., Seo T.S. (2022). An internet of things-based point-of-care device for direct reverse-transcription-loop mediated isothermal amplification to identify SARS-CoV-2. Biosens. Bioelectron..

[27] Park J., Woo S., Kim J., Lee H., Yoo Y.E., Hong S. (2021). Rapid and simple single-chamber nucleic acid detection system prepared through nature-inspired surface engineering. Theranostics.

[28] Xu Y., Yan H., Zhang Y., Jiang K., Lu Y., Ren Y., Wang H., Wang S., Xing W. (2015). A fully sealed plastic chip for multiplex PCR and its application in bacteria identification. Lab Chip.

[29] Ren P., Liu J., Zhao H., Fan X.P., Xu Y.C., Li C.X. (2019). Construction of a rapid microfluidic-based SNP genotyping (MSG) chip for ancestry inference. Forensic Sci. Int. Genet..

[30] Panpradist N., Wang Q., Ruth P.S., Kotnik J.H., Oreskovic A.K., Miller A., Stewart S.W.A., Vrana J., Han P.D., Beck I.A. (2021). Simpler and faster COVID-19 testing: Strategies to streamline SARS-CoV-2 molecular assays. EBioMedicine.

[31] Garcia-Venzor A., Rueda-Zarazua B., Marquez-Garcia E., Maldonado V., Moncada-Morales A., Olivera H., Lopez I., Zuniga J., Melendez-Zajgla J. (2021). SARS-CoV-2 Direct Detection Without RNA Isolation With Loop-Mediated Isothermal Amplification (LAMP) and CRISPR-Cas12. Front. Med..

[32] Chen J., Xu Y., Yan H., Zhu Y., Wang L., Zhang Y., Lu Y., Xing W. (2018). Sensitive and rapid detection of pathogenic bacteria from urine samples using multiplex recombinase polymerase amplification. Lab Chip.

[33] Zong N., Gao Y., Chen Y., Luo X., Jiang X. (2022). Automated Centrifugal Microfluidic Chip Integrating Pretreatment and Molecular Diagnosis for Hepatitis B Virus Genotyping from Whole Blood. Anal. Chem..

[34] Raja S., Ching J., Xi L., Hughes S.J., Chang R., Wong W., McMillan W., Gooding W.E., McCarty K.S., Chestney M. (2005). Technology for automated, rapid, and quantitative PCR or reverse transcription-PCR clinical testing. Clin. Chem..

[35] Cepheid GeneXpert® Infinity Systems. https://www.cepheid.com/en_US/systems/GeneXpert-Family-of-Systems/GeneXpert-Infinity..

[36] Senok A., Alfaresi M., Khansaheb H., Nassar R., Hachim M., Al Suwaidi H., Almansoori M., Alqaydi F., Afaneh Z., Mohamed A. (2021). Coinfections in Patients Hospitalized with COVID-19: A Descriptive Study from the United Arab Emirates. Infect. Drug Resist..

