# Supplementary material for: SMART: A Swing-Assisted Multiplexed Analyzer for Point-of-Care Respiratory Tract Infection Testing
Source: Biosensors (Basel). 2023 Feb 4;13(2):228. doi: 10.3390/bios13020228 (PMC9954503; doi:10.3390/bios13020228)
Supplement: Supplementary file 1 [file biosensors-13-00228-s001.zip › biosensors-2153446-supplementary.pdf]

Supporting Information

Supplemental information includes nine figures and five tables.

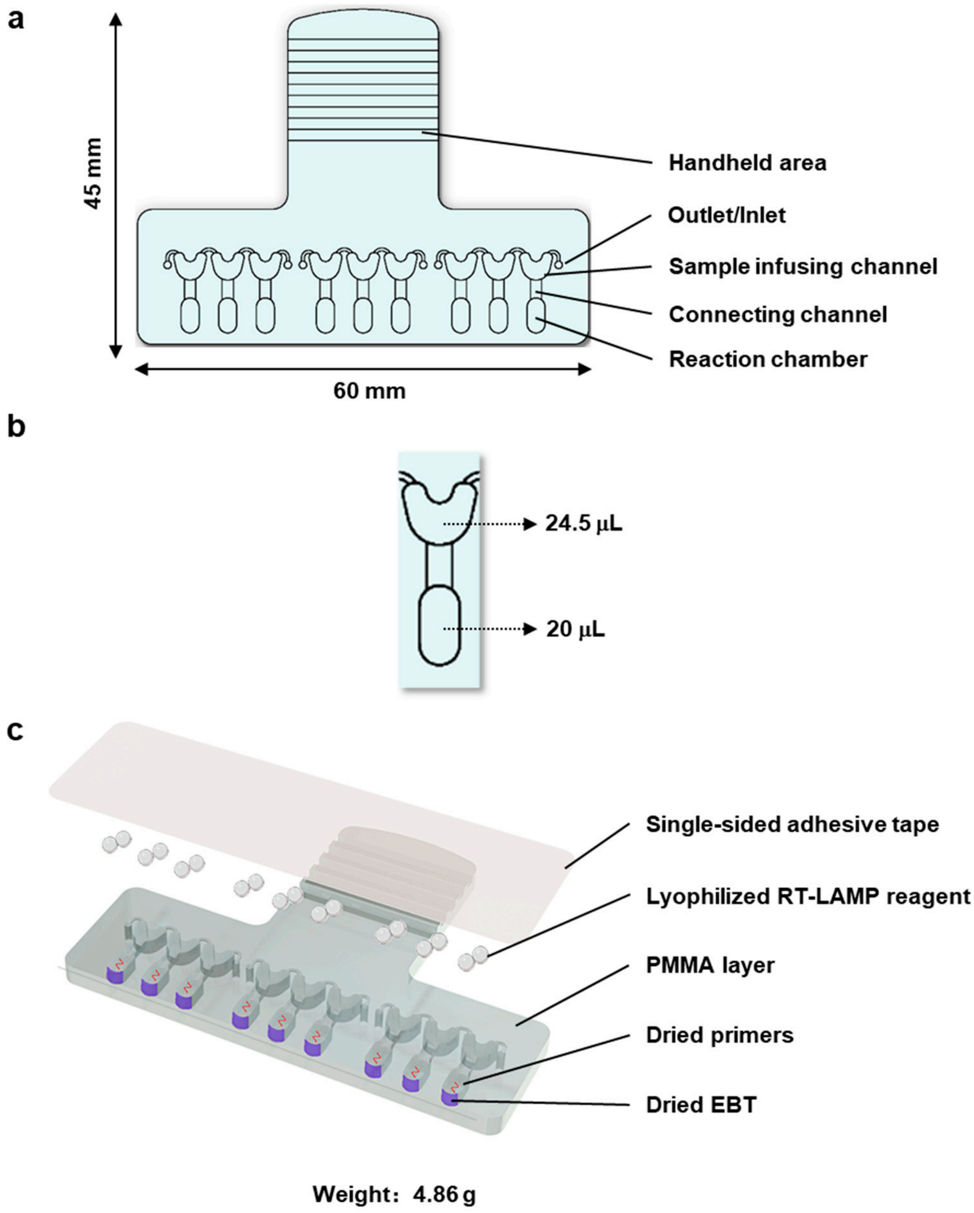

Figure S1. Design and assembly of the microfluidic chip. (a) Design and overall dimensions of the microfluidic chip. (b) Volume of each "valley" of reaction unit. Each reaction unit contains a "sine"-shaped sample infusing channel, three connecting channels, and three reaction chambers. The volume of each "valley" of the sample infusing channel was approximately 24.5 μL and the volume of the oval-shaped reaction chamber was approximately 20 μL. (c) An expanded view of the microfluidic chip assembly.

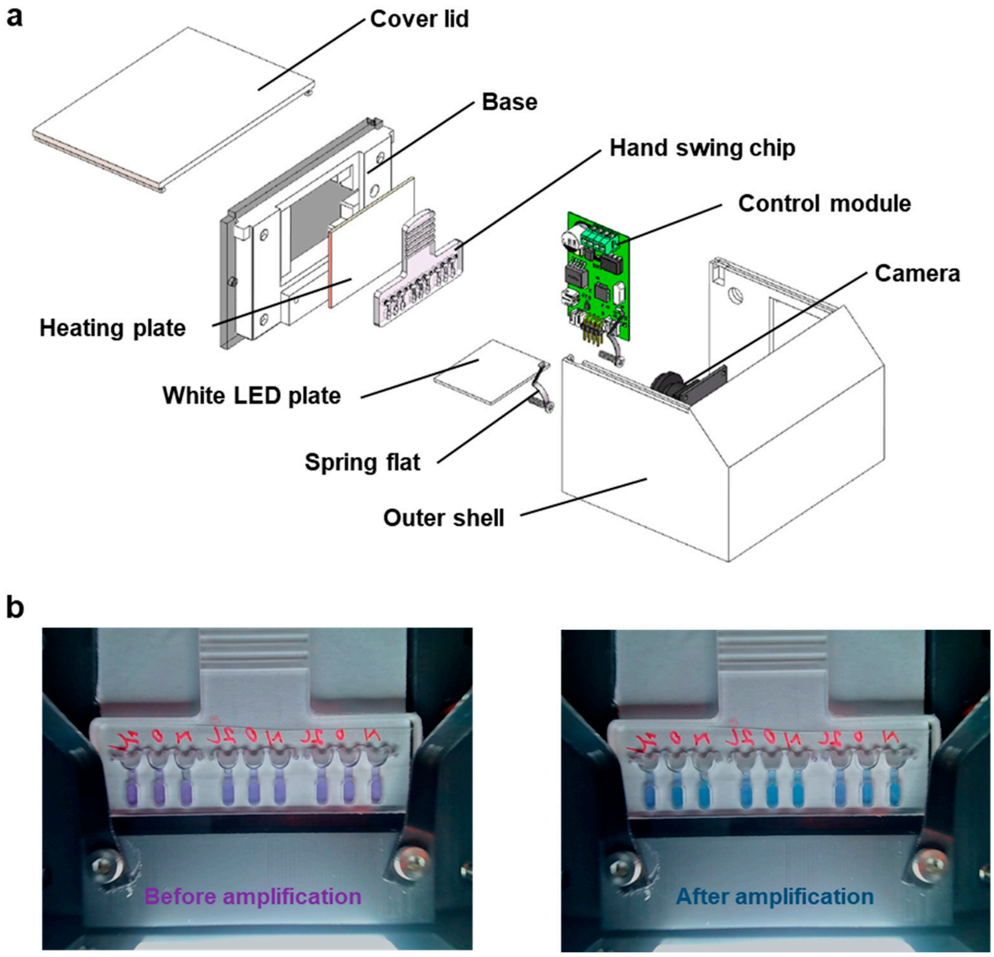

Figure S2. Construction of the analyzer. (a) An expanded view of the integrated analyzer. (b) A photograph of the chip compressed by two springs inside the instrument (left, before amplification; right, after amplification). The RGB value represents the color of the image. Due to the effect of EBT, the color for positive sample changes from purple to blue in the reaction represented by the significantly reduced in the R channel value and the significantly increased in the G channel value. Therefore, the value of k is defined as $\frac{G - R}{R + G + B}$ to reflect the reaction result.

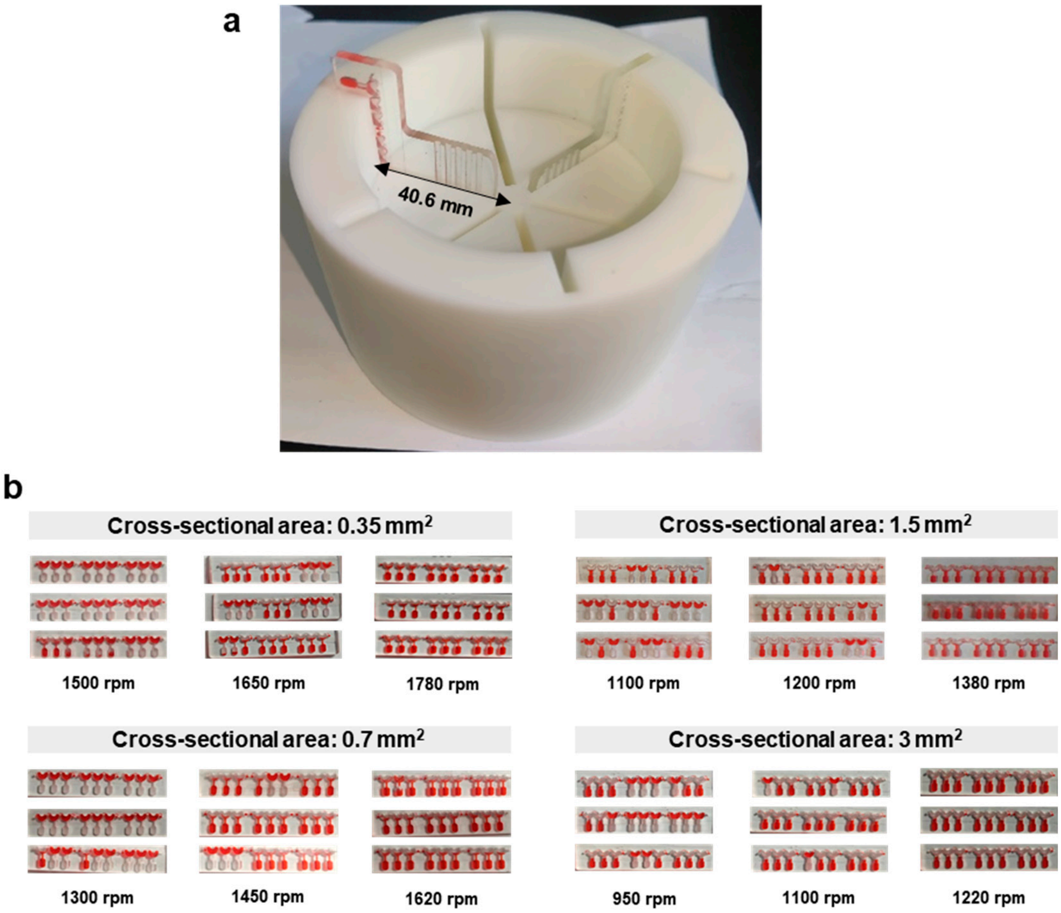

Figure S3. Optimization of the sizes of the connecting channel on the chip. (a) Centrifugal platform for holding the chips. (b) Liquid transfer at different connecting channels with varied cross-sectional areas at different centrifugal speeds.

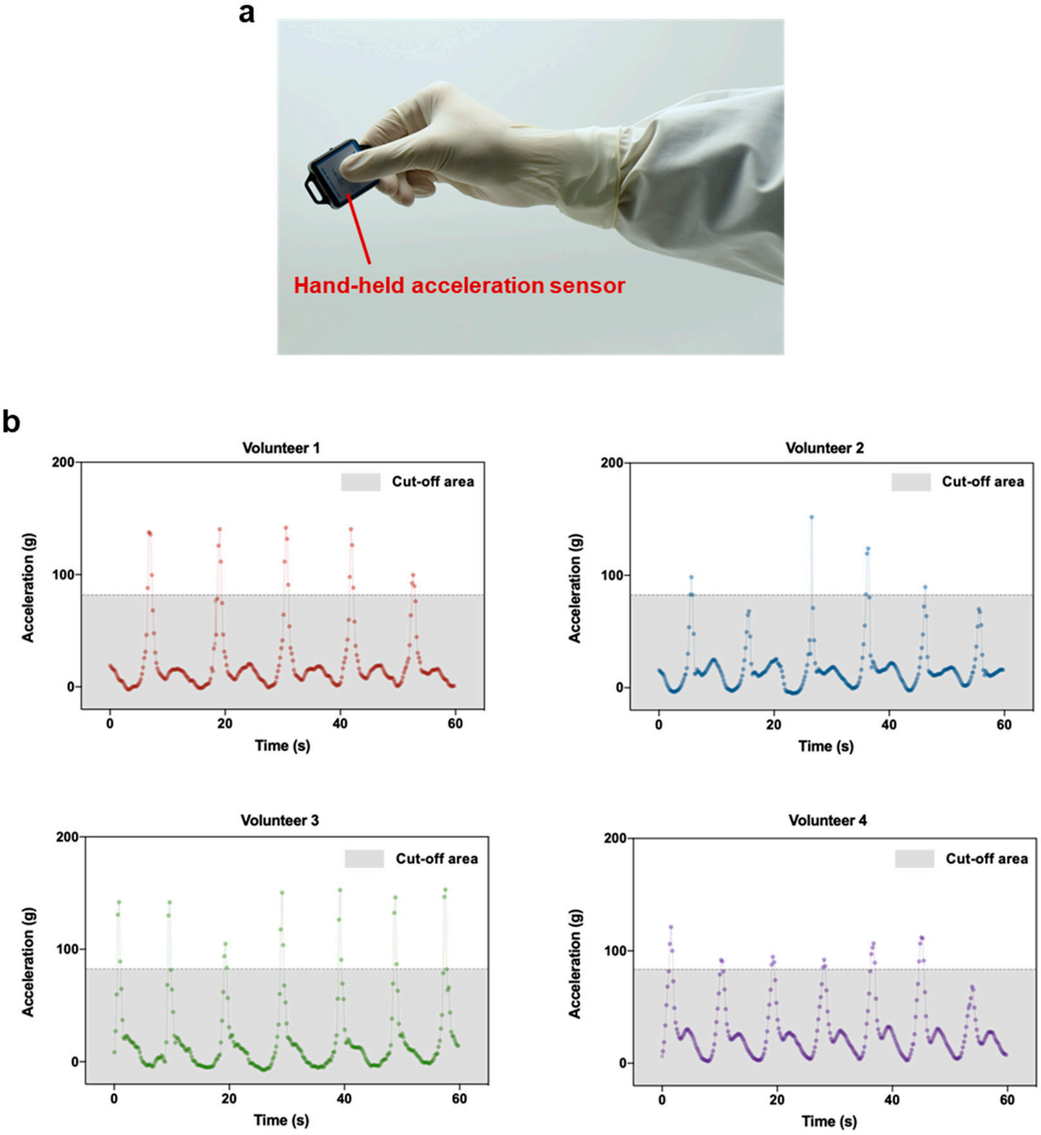

Figure S4. Statistics and measurement of the acceleration generated by hand-swinging. (a) A photograph of the hand-held accelerometer. (b) The acceleration of the hand swing in one minute by different volunteers. Volunteer 1 and 3 were males, volunteer 2 and 4 were females. The cut-off area indicates that acceleration in this region does not allow the liquid to be completely distributed.

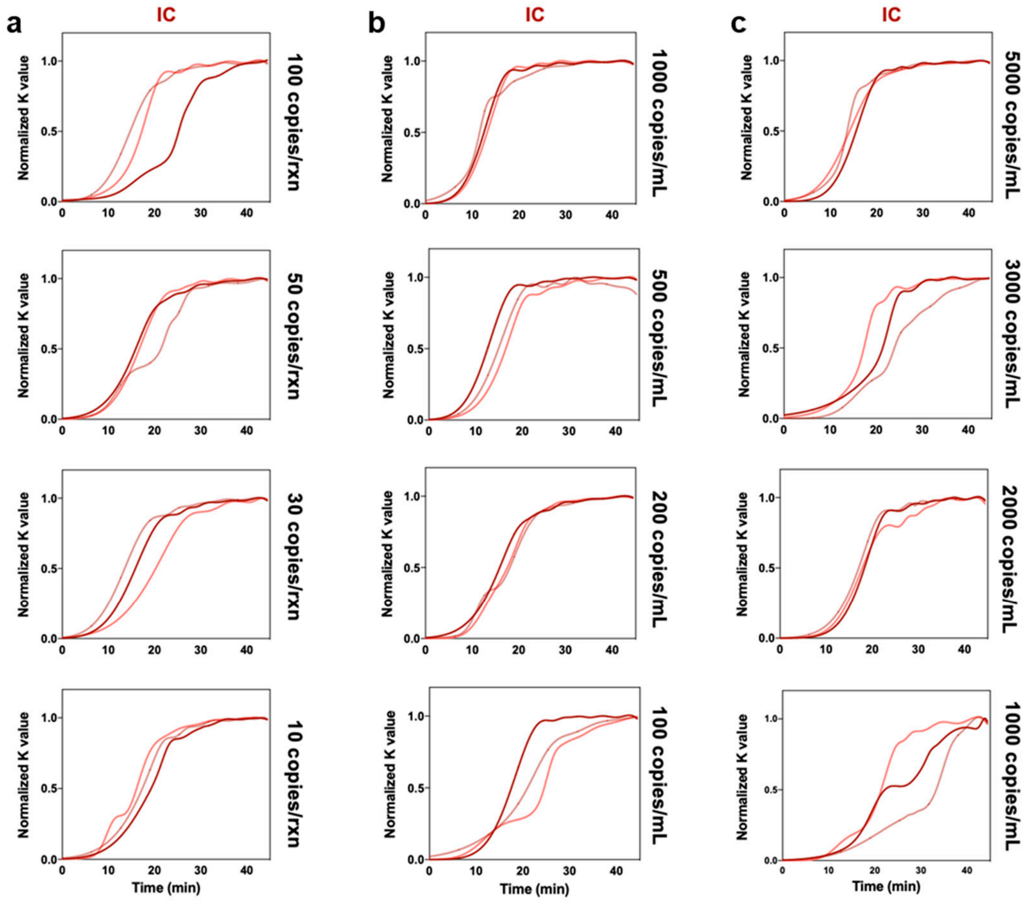

Figure S5. Real-time visualization of the IC detection results in the on-chip LoD test. (a) Test results of 20 ng human genomic DNA per reaction. (b) Test results with nucleic acid extracted from mock swab samples. (c) Test results with directly lysed raw mock swab samples. IC are well detected in all cases.

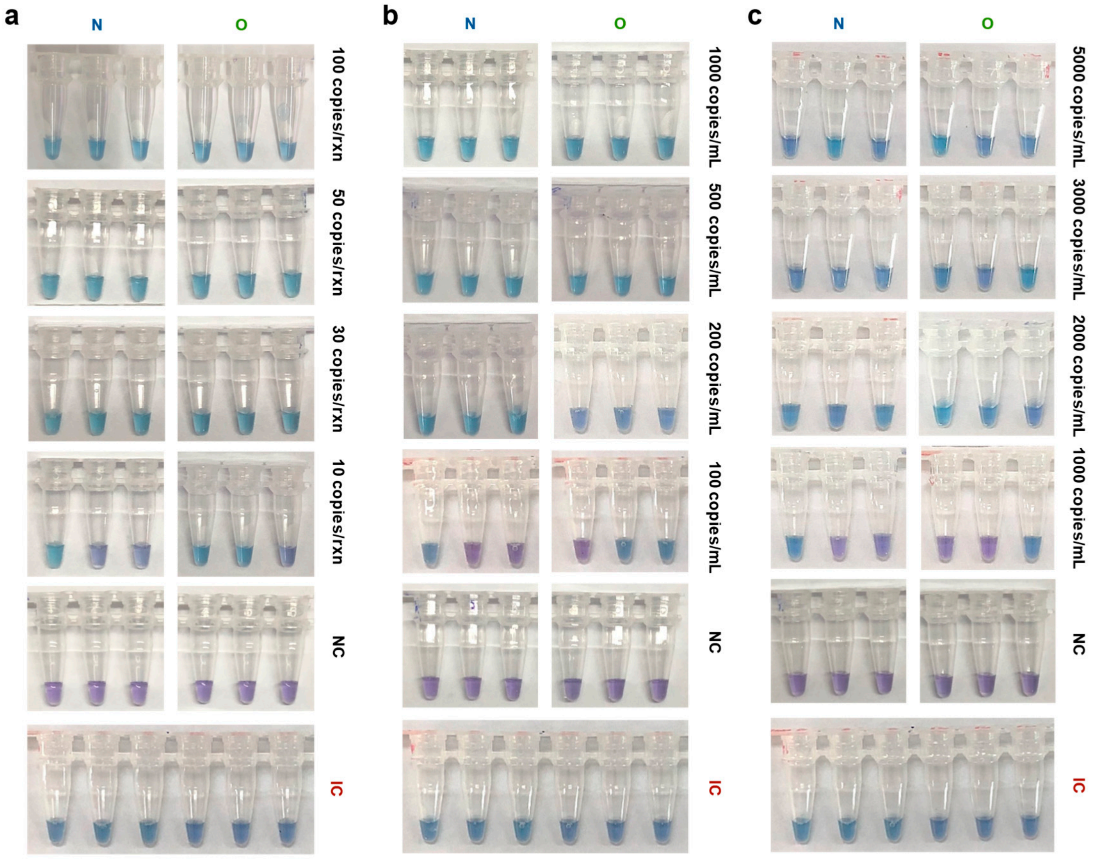

Copies/ reaction	10	30	50	100
N	1/3	3/3	3/3	3/3
O	2/3	3/3	3/3	3/3
IC	6/6			

Copies/ mL	100	200	500	1000
N	1/3	3/3	3/3	3/3
O	2/3	3/3	3/3	3/3
IC	6/6			

Copies/ mL	1000	2000	3000	5000
N	1/3	3/3	3/3	3/3
O	1/3	3/3	3/3	3/3
IC	6/6			

Figure S6. In-tube LoD test for SARS-CoV-2 detection. (a) End-point color change results of RT-LAMP reactions with synthetic SARS-CoV-2 viral RNA at concentrations of 10, 30, 50, and 100 copies/reaction. The IC reactions were performed with 20 ng of human genomic DNA per reaction. (b) End-point color change results of RT-LAMP reactions with NA extracted from SARS-CoV-2 mock swab samples with concentrations of 100, 200, 500, and 1000 copies/mL. (c) End-point color change results of RT-LAMP reactions with NA directly released from SARS-CoV-2 mock swab samples with concentrations of 1000, 2000, 3000, and 5000 copies/mL. The table attached summarizes the performance of sensitivity tested in-tube. (Note: NC, negative control. IC, internal control. rxn, reaction)

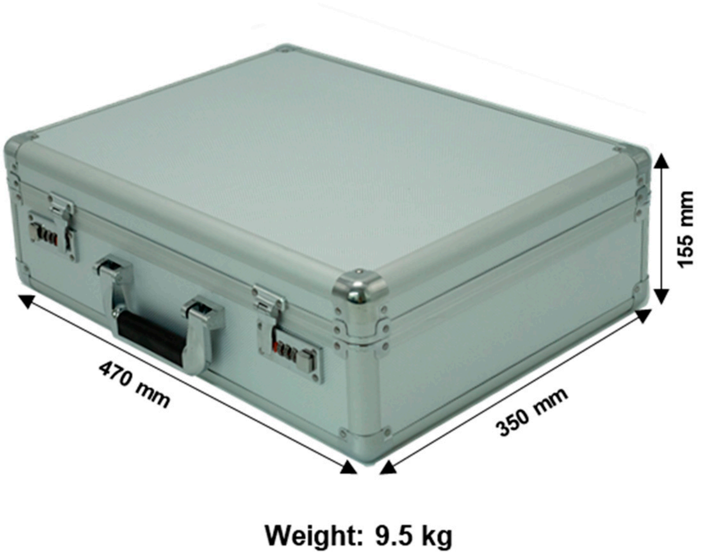

Figure S7. Dimensions and weight of the suitcase equipped with six SMART analyzers.

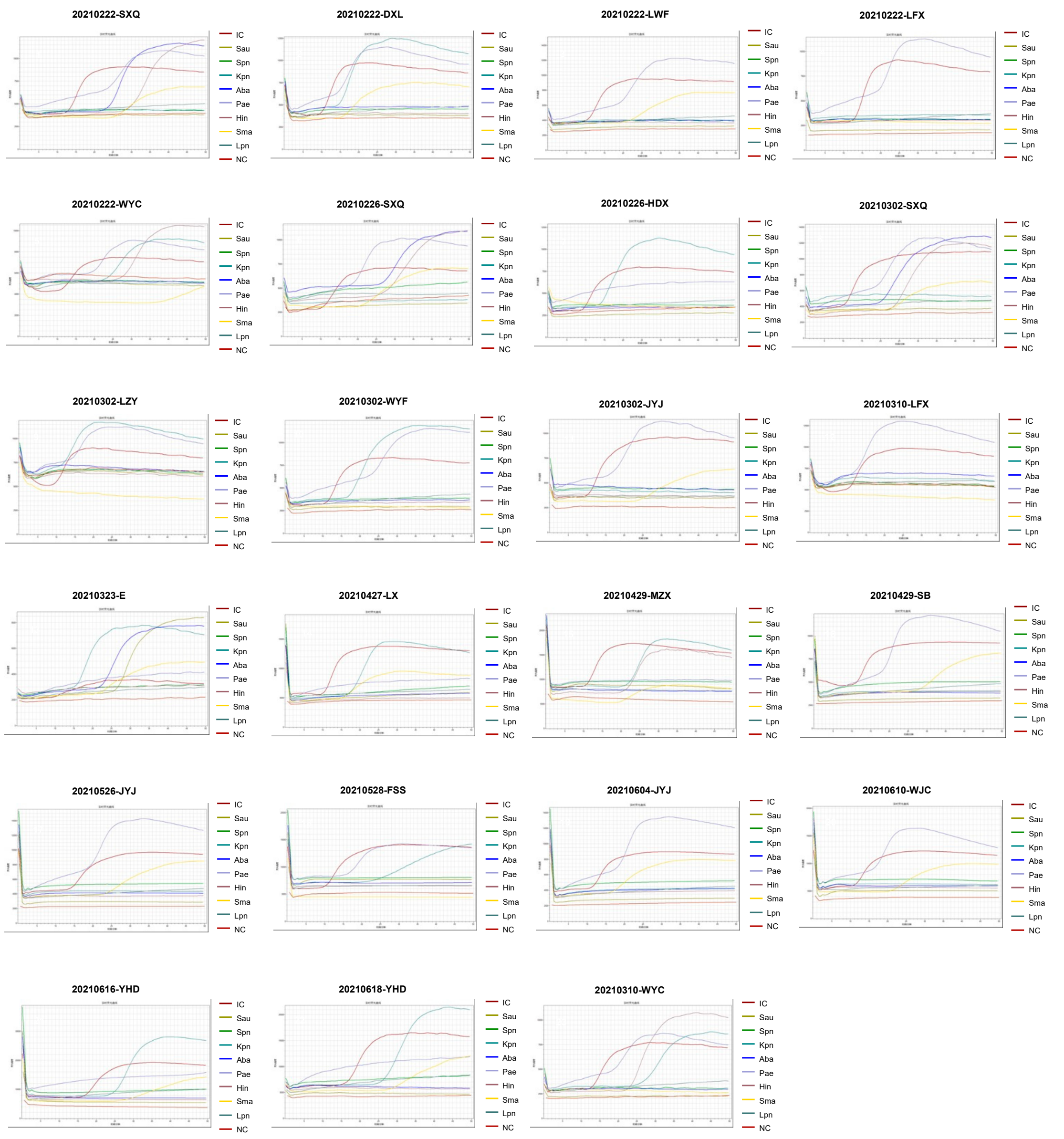

Figure S8. The results of 23 clinical sputum specimens analyzed by the commercial RTisochip™-A system. (Note: *Sau, Staphylococcus aureus. Spn, Streptococcus pneumoniae. Kpn, Klebsiella pneumoniae. Hin, Haemophilus influenzae. Aba, Acinetobacter baumannii. Pae, Pseudomonas aeruginosa. Sma, Stenotrophomonas maltophilia. Lpn, Legionella pneumophila*)

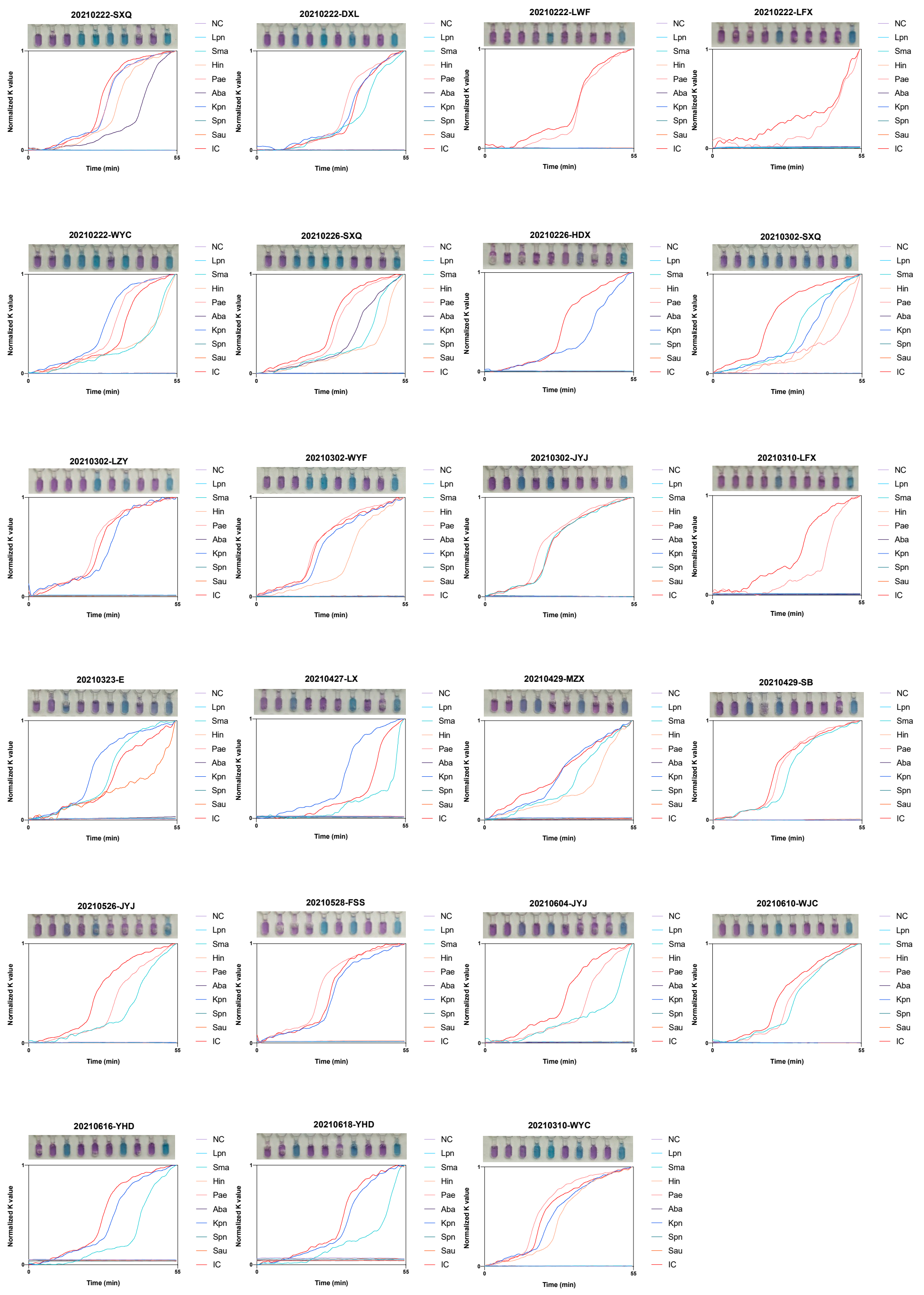

Figure S9. The results of 23 clinical sputum specimens analyzed by our SMART system. The real-time signal of each reaction is right plotted below with the image of the chip after the LAMP reaction was finished.

Table S1. Primer sequence of each target.

For SARS-CoV-2 detection:

Target		Primer	Sequence (5'-3')
SARS-CoV-2	N	F3	CCGCAAATTGCACAATTT
		B3	CCTTTTTAGGCTCTGTTGGTG
		FIP	GTGTAGGTCAACCACGTTCCCCTTCAGCGTTCTTCGGAAT
		BIP	TGCCATCAAATTGGATGACGTTTTGTATGCGTCAATATGCTT
		LF	GTGTGACTTCCATGCCAATGC
		LB	AAATTTCAAAGATCAAGTCATTTTGC
	O	F3	ATCGTGTTGTCTGTACTG
		B3	GTTCGCGGAGTTGATCACA
		FIP	CAAGTTGTAGGTATTTGTACATACGTTGCCACATAGATCATCCAAAT
		BIP	GACCCTGTGGGTTTTACACTTAATACAGCCATAACCTTTCCACATA
		LF	AGTCACAAAATCCTTTAG
		LB	ACAGTCTGTACCGTCTGC
IC		F3	GGACTCATGACCACAGTCCA
		B3	GCTTCCCGTTCAGCTCAG
		FIP	CGGCCATCACGCCACAGTTTCCATCACTGCCACCCAGA
		BIP	GGGGCTCTCCAGAACATCATCCGATGACCTTGCCCACAGC
		LF	GGAGGGGCCATCCACAGTC
		LB	GCCTCTACTGGCGCTGC

For respiratory bacteria detection:

Target	Primer	Sequence (5'-3')
IC	F3	CTTTAAAACTTGCTCTCTGCT
	B3	AGATCGGCTTGTTTCCTT
	FIP	CGGACAACTTTGGGACTCTAGGATTTAGTCTTAAATCGCTTCCTTCATG
	BIP	GCAAGGTGGGGAAACTGAACGATTTCAAAAAGGTACAACTCTCA
	LF	TCTAATCGGCGTGTTTGC

	LB	ATGTTTGCTTGCCAGTGCATTTTAG
***Staphylococcus aureus* (Sau)**	F3	GTGCCTTTACAGATAGCATG
	B3	GAAAAAGTGTACGAGTTCTTGA
	FIP	GTTTCATAACCTTCAGCAAGCTTTCCATACAGTCATTTCACGCA
	BIP	GAGGTCATTGCAGCTTGCTTACTTCGATCACTGGACCGCG
	LF	AACTCATAGTGGCCAACA
	LB	GTACCTGTTATGAAAGTGTTCA
***Streptococcus pneumoniae* (Spn)**	F3	AACTGATTGAAAGCCATTCA
	B3	ACGGCTAATGCCCCATT
	FIP	CAAACCTGCTTCATCTGCTAGAGTAGTCATGACGGACTACCGC
	BIP	CCGAAAACGCTTGATACAGGGAGTAACAACGGTCTGAGTGGTTGTTTGGT
	LF	TGCGTAAGAGTTCGATATAAAG
	LB	TTAGCTGGAATTAAAACGCACG
***Klebsiella pneumoniae* (Kpn)**	F3	GTCTCGCCAAGCGGAAT
	B3	ATGAGACCTACTGGGACA
	FIP	AACCGCAATGTGCAAAGCAGTCAGGGAGACGACCGT
	BIP	AGTTAATAAAGTAGGAGGCCTTGCTTTTGTTCGCGTATGGAATCGG
	LF	CGATGACAGCAGCAATAACCG
	LB	GCTATAGCCCACGCCCA
***Acinetobacter baumannii* (Aba)**	F3	TGTGCCAATTAACTTCTTAGC
	B3	CTTGTAATATCGTTATAGGCGTT
	FIP	AGTGAATTCGGTTATCGTAAGCTCGAAGCAGCAAAAAAAATTAGTCAC
	BIP	AATGATATGGCTCAAAAGCTAGAGGCTTAACTCATGTGCGATGG
	LF	TAGCAGAGAGGTCGCC
	LB	CCGTTAAAAATGCGCAGG
***Pseudomonas aeruginosa* (Pae)**	F3	TGTTATGGAAATGTCCACCTT
	B3	TCTGCTGAGCTTTCTGAG
	FIP	GGCCAGAACAGCAGCCAGAGGAACACGATGAACAACGT

	BIP	AGCTCGTCTGACCGCTACCCTTCGTCAGCCTTGCGAT
	LF	CCAGAGCAGAGAATTTCAGA
	LB	AGCTGCTCGTGCTCAGG
***Haemophilus influenzae* (Hin)**	F3	GCAGATGCAGTTAAAGGTT
	B3	GCTAATTGGTTAAATTACAAACGA
	FIP	ACCTAATACTGCAGGTTTTTCTTCAATCAACGGTAAAGGTGTTGATGCTGG
	BIP	GAAGCTGCATATTCTAAAAACCGTCGAAAAATGGATCCTGTTTTTCAAGT
	LF	CCGTAAGATACTGTGCCTAATT
	LB	GCAGTGTTAGCGTACTAATTCT
***Stenotrophomonas maltophilia* (Sma)**	F3	GTATAAGCTTGGAGGTTCCT
	B3	ATGAAGGCGGCCAGATG
	FIP	GCGCCAAGAGCTCAATAGTGGCACCGCTGATCAGGGA
	BIP	ACAACTAGTCGTTCACCGGTACGAGGCAATTACAAACGCGATG
	LF	GATGCCGGGGGCGGAA
	LB	ACCCGATGACAAGGGGCT
***Legionella pneumophila* (Lpn)**	F3	AATTCAATAAGAAAGCGGATGA
	B3	CGGTACTGTCAAAAACGGT
	FIP	GGCAATACAACAACGCCTGGAAAGTAAAAGGGGAAGCCT
	BIP	CTGGAAATGGTGTTAAACCCGGACCATCAATCAGACGACC
	LF	CTTGTTTTTGTTTTCAGTTAAAA
	LB	GGATACAGTCACTGTCGAATACA

Table S2. Maximum acceleration (g) per swing in one minute.

(Volunteer 1 and 3 were males, volunteer 2 and 4 were females)

Volunteer 1	Volunteer 2	Volunteer 3	Volunteer 4
137.661	98.657	141.919	121.2739
140.522	68.218	141.758	91.9644
141.855	151.968	104.863	94.7188
140.464	124.058	150.303	92.0821
99.741	89.653	152.788	106.7396
	70.063	146.167	112.0361
		153.184	68.0735

Table S3. Summary table for the detection of clinical respiratory pathogen samples.

	Sample information	IC	*Staphylococcus aureus* (Sau)	*Streptococcus pneumoniae* (Spn)	*Klebsiella pneumoniae* (Kpn)	*Acinetobacter baumannii* (Aba)	*Pseudomonas aeruginosa* (Pae)	*Haemophilus influenzae* (Hin)	*Stenotrophomonas maltophilia* (Sma)	*Legionella pneumophila* (Lpn)	NC
S01	**20210222-SXQ**	+			+	+	+	+			
S02	**20210222-DXL**	+			+		+		+		
S03	**20210222-LWF**	+					+				
S04	**20210222-LFX**	+					+				
S05	**20210222-WYC**	+			+		+	+	+		
S06	**20210226-SXQ**	+				+	+	+	+		
S07	**20210226-HDX**	+			+						
S08	**20210302-SXQ**	+			+		+	+	+		
S09	**20210302-LZY**	+			+		+				
S10	**20210302-WYF**	+			+		+	+			
S11	**20210302-JYJ**	+					+		+		
S12	**20210310-LFX**	+					+				
S13	**20210323-E**	+	+		+	+			+		
S14	**20210427-LX**	+			+				+		
S15	**20210429-MZX**	+			+			+	+		
S16	**20210429-SB**	+					+		+		
S17	**20210526-JYJ**	+					+		+		
S18	**20210528-FSS**	+			+		+				
S19	**20210604-JYJ**	+					+		+		
S20	**20210610-WJC**	+					+		+		
S21	**20210616-YHD**	+			+				+		
S22	**20210618-YHD**	+			+				+		
S23	**20210310-WYC**	+			+		+	+			

+ Positive signal

Table S4. The cost of the supporting analyzer.

Components	Model and brand	Cost
Camera	WXSJ-H65HD, Weixin, Shenzhen, China	$ 30
Control module	JLC, Shenzhen, China	$ 10
Base	Xmake, Shenzhen, China	$ 5
Outer shell		$ 4
Cover lid		$ 2
Heating plate	12 V, 5 W, Hongxin, Shenzhen, China	$ 0.30
LED panel light	Yingxin, Shenzhen, China	$ 0.20
Spring flat	Y3, Molitanhuang, Zhejiang, China	$ 0.02
In total		$ 51.52

Table S5. Comparison of existing molecular diagnostics with SMART system.

	Antigen test	Standard RT-qPCR	RT-qPCR-based POC systems	IA-based POC systems	SMART
**Target**	Protein	Nucleic acid	Nucleic acid	Nucleic acid	Nucleic acid
**POC(Y/N)**	Y	N	Y	Y	Y
**Method**	Lateral flow	PCR	PCR	IA	IA
**RNA extraction**	Not required	Required	Required	Not required	Not required
**Turnaround time**	<20 min	75-120 min	30-120 min	<60 min	<60 min
**Apparatus reliance**	Low	High (RT-qPCR machine)	High (GeneXpert/ FilmArray)	Medium (Servo/motor)	Medium
**Equipment cost**	-	>$45,000	>$32,000	<$1000	<$100
**User requirement**	Short-trained	Well-trained	Short-trained	Short-trained	Short-trained
**Mass on-site screening**	Not feasible	Not feasible	Feasible	Feasible	Feasible
**Result readout**	Easy	Not easy	Easy	Easy	Easy

IA: isothermal amplification
